# Supplementary material for: Biological Activities and Chemical Profiles of Kalanchoe fedtschenkoi Extracts
Source: Plants (Basel). 2023 May 10;12(10):1943. doi: 10.3390/plants12101943 (PMC10223013; doi:10.3390/plants12101943)

Supplementary information

Biological Activities and Chemical Profiles of *Kalanchoe fedtschenkoi* extracts

Jorge L. Mejía-Méndez [1,*], Horacio Bach [2], Ana C. Lorenzo-Leal [2], Diego E. Navarro-López [3], Edgar R. López-Mena [3], Luis Ricardo Hernández [1], and Eugenio Sánchez-Arreola [1,*]

[1] Laboratory in Phytochemistry Research, Chemical Biological Sciences Department, Universidad de las Américas Puebla, Ex Hacienda Sta. Catarina Mártir S/N, San Andres Cholula 72810, Mexico; luisr.hernandez@udlap.mx

[2] Division of Infectious Diseases, Faculty of Medicine, University of British Columbia, Vancouver, BC V6G 3Z6, Canada; hbach@mail.ubc.ca (H.B.); anacecylole@gmail.com (A.C.L.-L.)

[3] Tecnologico de Monterrey, Escuela de Ingeniería y Ciencias, Campus Guadalajara, Av. Gral. Ramón Corona No 2514, Colonia Nuevo México, Zapopan, 45121 Mexico; diegonl@tec.mx (D.E.N.-L.); edgarl@tec.mx (E.R.L.-M.)

* Correspondence: jorge.mejiamz@udlap.mx (J.L.M.-M.); eugenio.sanchez@udlap.mx (E.S.-A.)

Figure S1. GC/MS chromatogram of hexane extract from *K. fedtschenkoi*

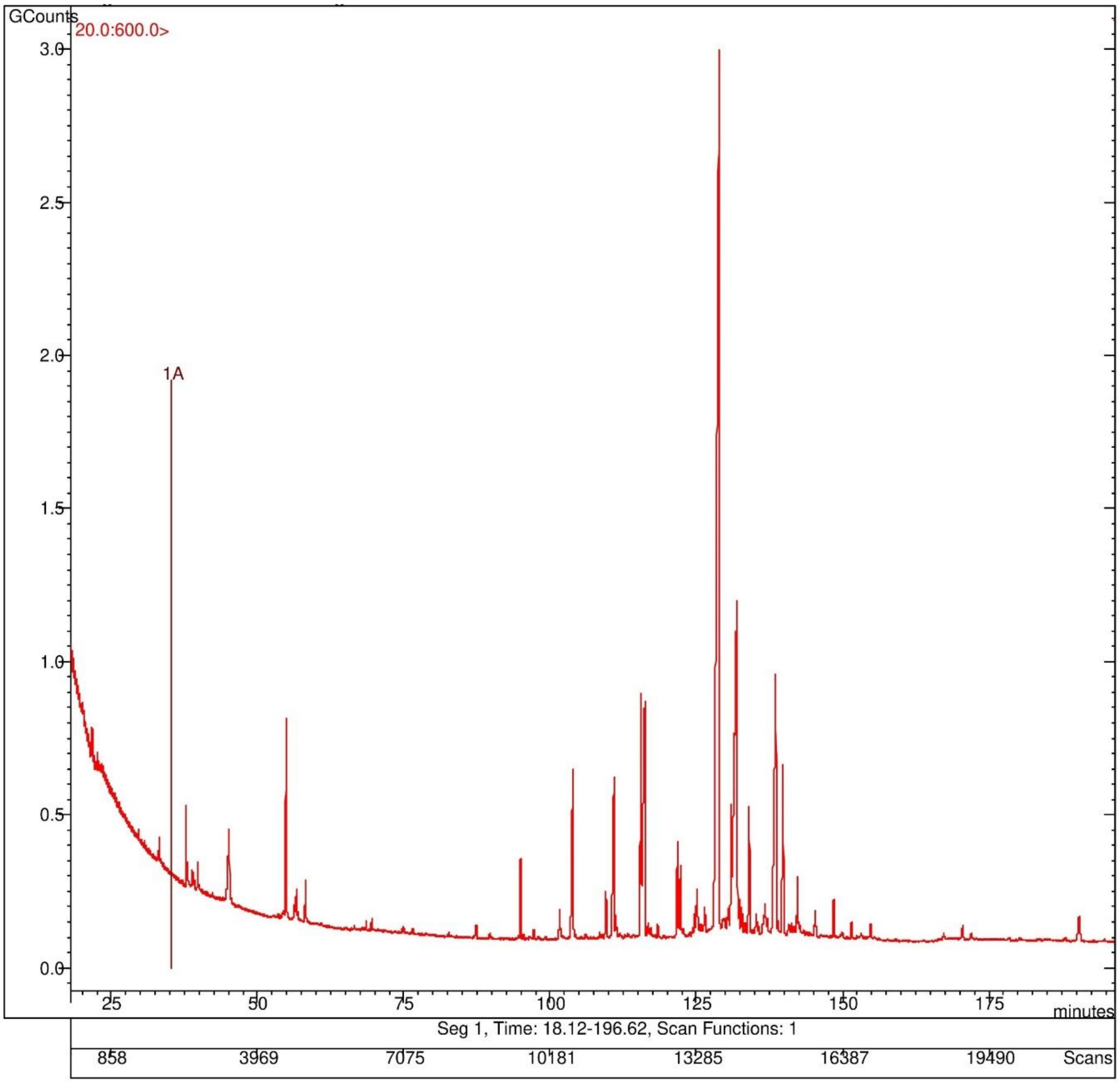

Figure S2. GC/MS chromatogram of chloroform extract from *K. fedtschenkoi*.

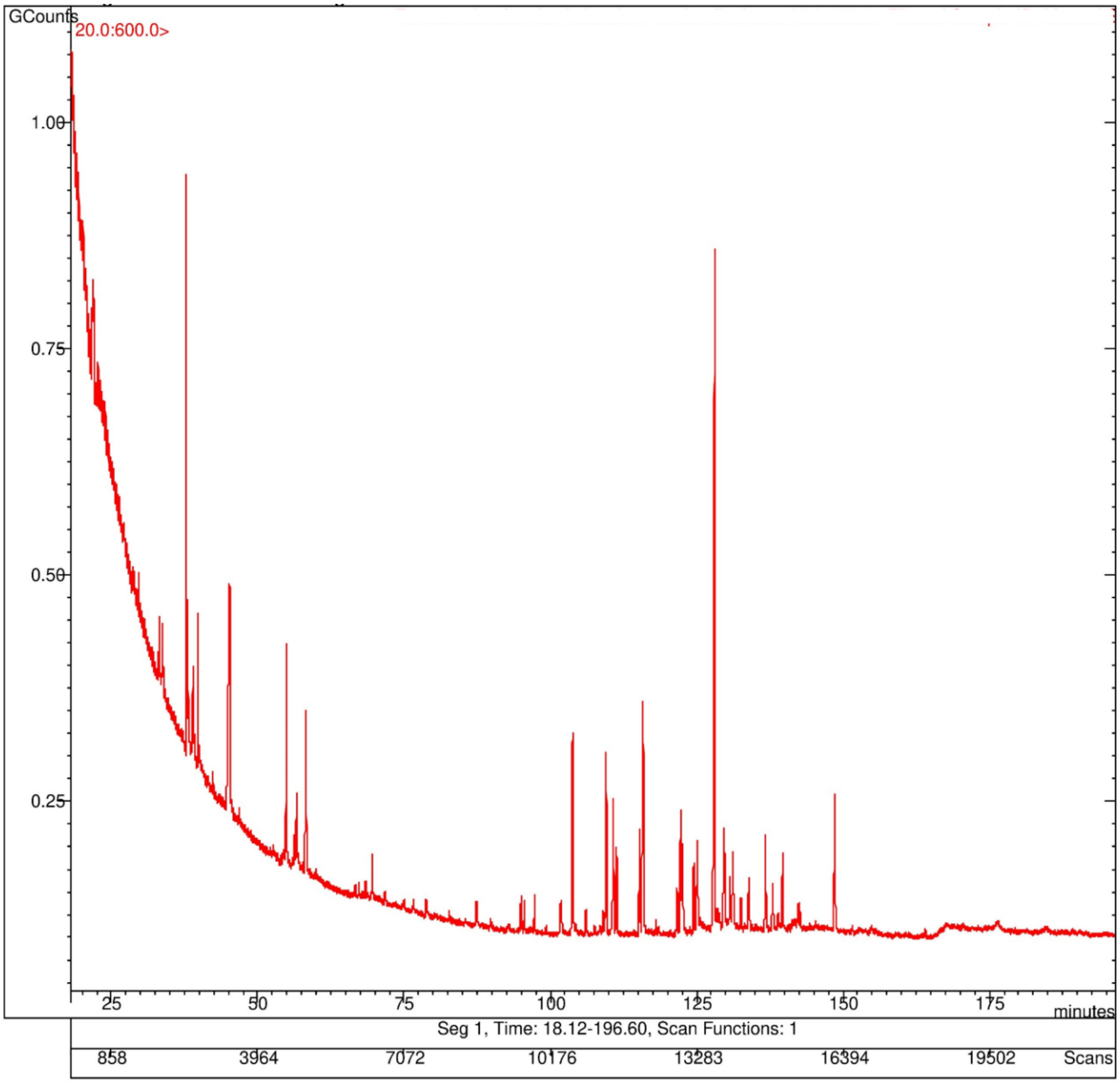

Figure S3. HPLC chromatogram of methanol extract from *K. fedtschenkoi*.

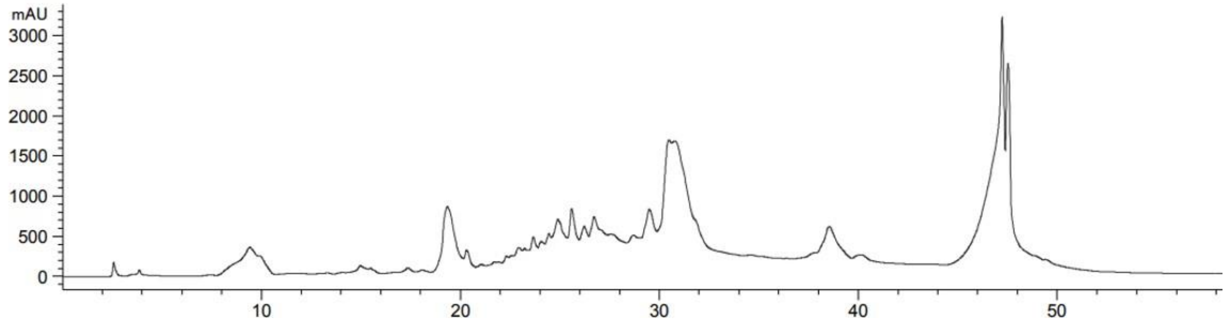

Figure S4. Calibration curve of quercetin used as standard solution.

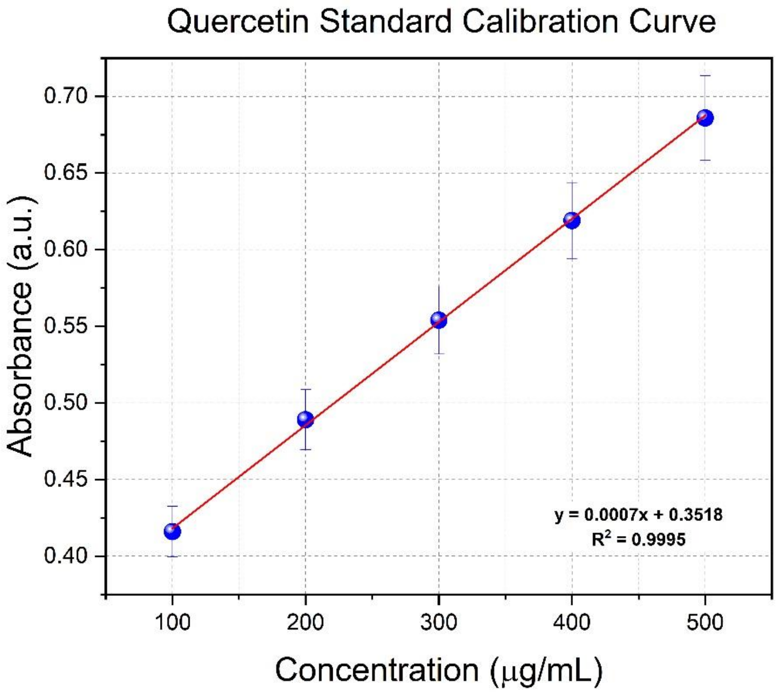

Supplement: Supplementary file 1 [file plants-12-01943-s001.zip › plants-2341318-supplementary.pdf]
